# Sponge non-metastatic Group I Nme gene/protein - structure and function is conserved from sponges to humans

**DOI:** 10.1186/1471-2148-11-87

**Published:** 2011-04-01

**Authors:** Drago Perina, Maja Herak Bosnar, Ružica Bago, Andreja Mikoč, Matija Harcet, Martina Deželjin, Helena Ćetković

**Affiliations:** 1 Division of Molecular Biology, Ruđer Bošković Institute, Bijenička cesta 54, 10002 Zagreb, Croatia; 2 Division of Molecular Medicine, Ruđer Bošković Institute, Bijenička cesta 54, 10002 Zagreb, Croatia

## Abstract

**Background:**

Nucleoside diphosphate kinases NDPK are evolutionarily conserved enzymes present in Bacteria, Archaea and Eukarya, with human Nme1 the most studied representative of the family and the first identified metastasis suppressor. Sponges (Porifera) are simple metazoans without tissues, closest to the common ancestor of all animals. They changed little during evolution and probably provide the best insight into the metazoan ancestor's genomic features. Recent studies show that sponges have a wide repertoire of genes many of which are involved in diseases in more complex metazoans. The original function of those genes and the way it has evolved in the animal lineage is largely unknown. Here we report new results on the metastasis suppressor gene/protein homolog from the marine sponge *Suberites domuncula*, NmeGp1Sd. The purpose of this study was to investigate the properties of the sponge Group I Nme gene and protein, and compare it to its human homolog in order to elucidate the evolution of the structure and function of Nme.

**Results:**

We found that sponge genes coding for Group I Nme protein are intron-rich. Furthermore, we discovered that the sponge NmeGp1Sd protein has a similar level of kinase activity as its human homolog Nme1, does not cleave negatively supercoiled DNA and shows nonspecific DNA-binding activity. The sponge NmeGp1Sd forms a hexamer, like human Nme1, and all other eukaryotic Nme proteins. NmeGp1Sd interacts with human Nme1 in human cells and exhibits the same subcellular localization. Stable clones expressing sponge NmeGp1Sd inhibited the migratory potential of CAL 27 cells, as already reported for human Nme1, which suggests that Nme's function in migratory processes was engaged long before the composition of true tissues.

**Conclusions:**

This study suggests that the ancestor of all animals possessed a NmeGp1 protein with properties and functions similar to evolutionarily recent versions of the protein, even before the appearance of true tissues and the origin of tumors and metastasis.

## Background

The Nme family, initially called nucleoside diphosphate kinases (NDPK) or Nm23, are evolutionarily conserved proteins present in all three domains of life: Bacteria, Archaea and Eukarya [1]. Vertebrate Nme enzymes can be separated into two evolutionarily distinct groups. In humans, Group I includes Nme1-Nme4 and Group II includes Nme5-Nme9 proteins. Nme10, also known as XRP2, was the last described member and apparently has a somewhat different evolutionary history to Group I or Group II genes; it is characterized by a recent insertion of a partial NDPK domain [2]. The human Nme1 was recognized as the first metastasis suppressor and is the most studied member of the Nme family of proteins [3]. In contrast to tumor suppressor genes, metastasis suppressor genes do not abolish or diminish the tumorigenicity of a tumor, they only affect its potential to metastasise. This means that a metastasis suppressor fulfills its biological function within the processes linked to the metastatic cascade: tumor cell dissociation, invasion of the surrounding tissue, journey through the blood circulation, invasion and secondary tumor growth at a distinct site in the body [4]. In addition to melanoma [5], the metastasis suppressor activity of Nme1 has been detected in breast [6], cervical [7], hepatocellular carcinoma [8], and several other neoplastic lesions [5]. The Nme1/NDPK A and Nme2/NDPK B protein products represent two subunits of a well known, house-keeping enzyme - nucleoside-diphosphate kinase (NDPK). These subunits can assemble into the enzymatically active hexamer in all possible combinations (A6, A5B....... AB5, B6) [9]. NDPK is involved in the maintenance of the cellular NTP pool, transferring the phosphate group through the histidine phosphointermedier. Interestingly, it seems that this biochemical feature of NDPK is not responsible for its antimetastatic activity [10]. Subsequently, several other biochemical functions have been assigned to this protein: histidine kinase activity [11,12], 3'-5' exonuclease and DNA cleavage activity [13], and transcriptional regulatory activity [14]. The Nme family is known to participate in numerous crucial biological events such as proliferation [15], differentiation [16,17], development [18,19] and apoptosis [20,21], as well as in adhesion, migration [22], and vesicular trafficking [23]. In spite of the comprehensive scientific activity in this area it is still unclear which biochemical/biological activities are responsible for Nme's antimetastatic role.

A recent evolutionary study on all known vertebrate Nmes showed that *Nme1 *and *Nme2 *arose by cis-duplication [2]. Nme1 and Nme2 proteins are 88% identical in amino-acid sequences and their genes are located next to each other. Although very similar in primary structure, Nme1 and Nme2 are well distinguished by their distinct pI value: 5.8 and 8.5, respectively. Cis-duplication and the appearance of *Nme1 *probably occurred in a common ancestor of amniotes [2]. However, Bilitou et al. [1] observed two NmeGp1 sequences in the cnidarian *Nematostella vectensis *also located next to each other, and Desvignes et al. [24] found independent duplications of *NmeGp1 *genes in several opisthokont lineages.

Sponges (Porifera) are the simplest metazoan phylum and branched off first from the common ancestor of all animals [25,26], however, see also [27,28] for an alternative view. They have changed little during evolution and can provide insight into the metazoan ancestor's genomic features [29-33]. The genome of the marine sponge *Suberites domuncula *encodes at least two Nme proteins. One belongs to Group I and displays a high similarity to its human Nme1 homolog (Nm23-H1), therefore it was named Nm23-SD1 when it was first described [34]. In accordance with the new nomenclature, this protein is now renamed to NmeGp1Sd. The other protein, Nme6Sd (previously Nm23-SD6) is most similar to human Group II member Nme6 protein (Nm23-H6) [34]. The total number of Nme proteins in *S. domuncula *is possibly greater than two because the EST database used in the *S. domuncula *Nme study [34] contained at most 4500 non-redundant cDNA sequences which is certainly far less than the total number of genes in the *S. domuncula *genome. Orthologues of human Nme5, Nme6 and Nme7 appeared very early in animal evolution and are present in basal metazoans - cnidarian *N. vectensis *and placozoan *T. adhaerens *as well as in choanoflagelate *M. brevicollis *genomes. Group I Nme diversified much later, after the appearance of chordates [1,24]. Therefore, we expect that most of the Nme enzymes in sponges are probably members of Group II.

Although the sponge *S. domuncula *NmeGp1Sd protein displays high similarity with vertebrate Nme1, the most recent Group I member, it may reflect characteristics of the protein ancestral to Group I, before the later duplications and diversifications that have occurred within this Group. Our goal was to determine the gene structure and study the biochemical characteristics and functions of the *S. domuncula *protein NmeGp1Sd. Furthermore, we wanted to test whether it can substitute for the most recent (and the most studied) Group I member human Nme1, and show a similar ability to suppress migration and, therefore, possibly even the metastatic potential of human cancer cells *in vitro*.

## Results

### Structure and evolution of sponge Group I Nme gene

Group I Nme genes were analyzed in two sponges: *Amphimedon queenslandica *and *Suberites domuncula*. Only one gene coding for Group I Nme enzyme was found in the genome of the sponge *A. queenslandica*. A search of the corresponding EST database revealed the presence of two cDNA types with 5'untranslated regions (UTRs) 63 bp and 76 bp long, and therefore the existence of two transcription initiation sites. The 5'UTR region of *NmeGp1Sd *is 18 bp long and the transcription start site is located after oligopyrimidine tract. In *A. queenslandica *the TATA box-like motif (CCTATCAGCT) was identified in a region -33 to -24 nucleotides upstream from the transcriptional start of the shorter EST. In *S. domuncula *the *NmeGp1Sd *gene TATA box like motif (TCTAGAAATT) was found spanning from -81 to -72 nucleotides. The human *Nme1 *gene has no TATA box. It contains a number of motifs which may bind known transcriptional regulatory proteins such as AP-1, CTF/NF1, ACAAAG, and Ets [35]. An Ets binding sequence was found spanning from -70 to -61 nucleotides (ACCGGACGCC) in the promoter region of the *NmeGp1 *gene from sponge *A. queenslandica *and from -365 to -374 nucleotides (ACAGGATATA) in the promoter region of the *NmeGp1Sd *gene from sponge *S. domuncula*. Chen et al. [36] showed that the presence of a motif typical for AP-1 transcriptional elements in the 5' region of the human *Nme1 *gene is essential for promoter activity. A putative site for AP-1 in the *A. queenslandica NmeGp1 *5' flanking region was found spanning from -117 to -125 nucleotides (TTGACTCAG) and in *S. domuncula NmeGp1 *from -130 to -138 nucleotides (ATGTCTCAG). The structure of sponge *NmeGp1 *promoter regions is shown in Additional file 1.

**Figure 1:**
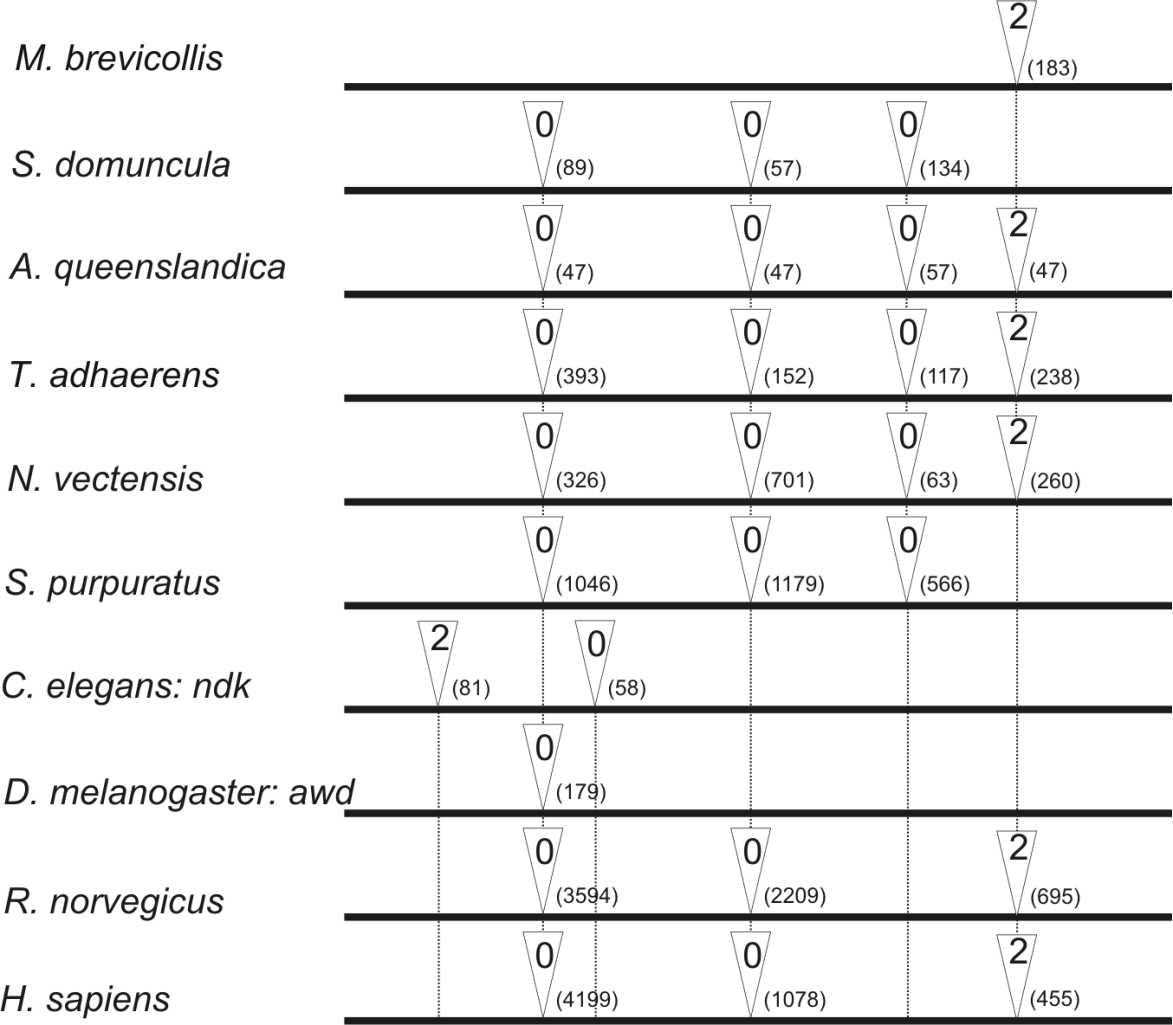
**Intron-mapping of *NmeGp1 *genes from representative species**. Triangles indicate positions of introns. The number in the triangle denotes the phase: 0, intron insertion between two triplet codons; 1, intron insertion after the first base of a codon; 2, intron insertion after the second base. Intron lengths are shown in brackets.

The *Nme *gene from *A. queenslandica *is 654 bp long from the ATG start codon to the TAG stop codon. It has four short introns with lengths of 47, 47, 57 and 47 bp, respectively. The *NmeGp1Sd *gene from *S. domuncula *coding region and introns encompasses 736 bp from the ATG start codon to the TAA stop codon and contains three introns (89, 57 and 134 bp). Their positions and phases are shown in Figure 1. Multiple sequence alignment of *NmeGp1Sd *gene homologs was produced and used to compare intron positions. The results are shown in Figure 1. *NmeGp1Sd *gene lacks the fourth intron which is present in the homolog from *A. queenslandica*.

As mentioned before, two *NmeGp1 *genes in the cnidarian *N. vectensis *were found. Like *Nme1 *and *Nme2 *in mammals and lizard, these two genes are located next to each other. Therefore, we checked neighboring genes in the genome of *A. queenslandica *and found that the *NmeGp1 *gene is surrounded by *unc50 *and *acad8 *genes and not by another *Nme *homolog. Extensive searches of the *A. queenslandica *genome [32] and *S. domuncula *cDNAs [33] revealed only one *NmeGp1 *gene in each of these two demosponges. Independent duplications have also been identified in *Branchiostoma floridae *and *Ciona intestinalis *[2] as well as more recently in other opisthokonts [24]. Searches of NCBI's dbEST revealed the presence of cDNAs that encode two NmeGp1 proteins in the calcareous sponges *Sycon raphanus *[GenBank:AM764253 and GenBank:AM764213] and *Leucetta chagosensis *[GenBank:GO094744 and GenBank:GO094741]. Phylogenetic analysis shows that distinct sponge proteins from the same species clearly group together, indicative of independent duplications (Additional file 2). The cnidarian *Hydra magnipapillata *also has at least two Group I member genes which are not located next to each other in the genome. Demosponge proteins cluster in a poorly supported and poorly resolved branch together with cnidarian, placozoan, *S. purpuratus *and *B. floridae *homologs while calcarean proteins form a separate branch.

### The sponge NmeGp1Sd protein forms a hexamer

**Figure 2:**
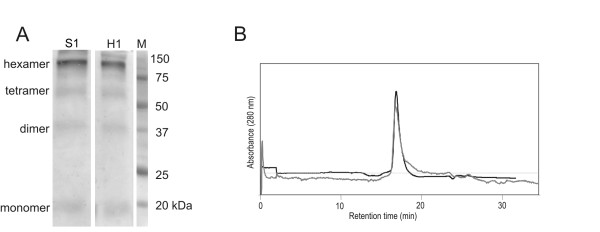
**Oligomerization of sponge NmeGp1Sd protein and human Nme1 as control**. (A) Cross-linking NmeGp1Sd (S1) and Nme1 (H1) with glutaraldehyde. Purified recombinant protein NmeGp1Sd (8 μg) was pre-incubated in PBS at room temperature with 25 mM of glutaraldehyde to initiate the cross-linking. After quenching, the reaction product was subjected to 12.5% SDS-PAGE gel followed by staining with Coomassie brilliant blue. (B) Size exclusion chromatography. Recombinant human Nme1 and sponge NmeGp1Sd were loaded onto Bio-Sil SEC 250 gel-filtration columns (300 mm × 7.8 mm) and eluted with Nm23 buffer at a flow rate of 0.5 mL/min. Black line represents human Nme1 and grey line sponge NmeGp1Sd protein.

Various oligomeric structures such as hexamers, tetramers, dimers, and monomers were found after cross-linking the NmeGp1Sd protein with glutaraldehyde. Recombinant NmeGp1Sd appears to be predominantly in the hexameric form (Figure 2A). A similar oligomeric pattern was observed with the recombinant human Nme1 protein. The hexameric form was expected due to the conservation of the KPN loop (residues 91 to 113 in NmeGp1Sd). Each KPN loop interacts with two others with helices α1 and α3, and with the four terminal residues [1], well preserved in the sponge NmeGp1Sd (residues 148 to 151). Residues Lys 30, Pro 95 [37] and Ser 119 [38] known to be crucial for hexamer formation are also conserved in the sponge NmeGp1Sd (Additional file 3). Thus, we can conclude that all the residues necessary for the hexameric structure are present in the sponge NmeGp1Sd enzyme, and known residues that could impede this structure are absent. The hexameric structure of sponge NmeGp1Sd was also confirmed by gel filtration (Figure 2B). Previously described human Nme1 [39] was used as marker. Overlapping gel filtration peaks confirm our finding that both proteins have a similar molecular weight as well as hexameric structure.

### The sponge NmeGp1Sd protein has a similar level of kinase activity as the human Nme1 but shows nonspecific DNA-binding activity

The specific kinase activities of the sponge NmeGp1Sd and human Nme1 enzymes were measured in parallel and found to be 496 U/mg and 407 U/mg, respectively. In comparison, Ma et al. [13] found the kinase activity of Nme1 protein to be 570 U/mg while Bago et al. [39] observed 237 U/mg. The specific activities of the sponge NmeGp1Sd enzyme and human Nme1 kinase were similar and within the previously reported ranges for the human protein.

**Figure 3:**
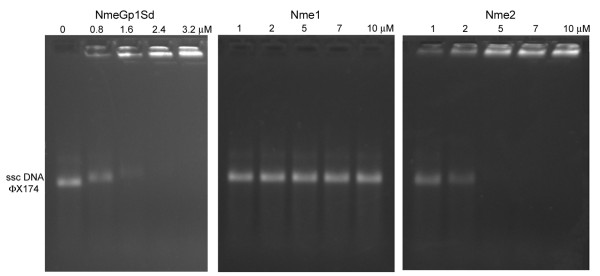
**DNA-binding activity of the NmeGp1Sd protein**. Human Nme1 and Nme2 proteins were used as control. The reaction was performed in buffer containing 40 mM Tris-acetate (pH 7.5) and 12 mM MgCl_2_, 30 nM of single-stranded circular (ssc) DNA from bacteriophage ϕX174 and purified NmeGp1Sd protein as indicated. 40 mM Tris-acetate (pH 7.5) containing 50% glycerol and 0.01% bromophenol blue was added before the products were analyzed in 0.6% agarose gel.

Purified NmeGp1Sd protein is able to bind non-specifically to single-stranded circular DNA (sscDNA) (Figure 3). Binding activity was observed with concentration of 0.8 μM, manifested by increase in DNA band retardation. The majority of the protein-DNA complexes at concentrations of NmeGp1Sd >1.7 μM exist in the form of big aggregates which stack in the wells (Figure 3, left). Human Nme1 did not display an ability to bind sscDNA, while human Nme2 binds sscDNA and shows the same DNA band retardation effect as sponge NmeGp1Sd. However, higher concentration (>4 μM) of human Nme2 protein was needed to form the same amount of aggregates (Figure 3, right).

### The sponge NmeGp1Sd does not cleave negatively supercoiled DNA

**Figure 4:**
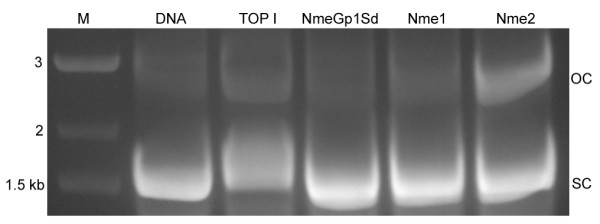
**Cleavage of negatively supercoiled pUC19-NHE plasmid DNA**. Reaction was assembled in 50 mM KCl, 10 mM MgCl_2_, 50 mM Tris HCl pH 7.5, 0.5 mM DTT, 30 μg/mL BSA with 500 ng of pUC19-NHE plasmid DNA (lane DNA) and 500 ng of proteins topoisomerase I, NmeGp1Sd, human Nme1 and Nme2, as indicated. After 1% SDS, 10 mM EDTA and proteinase K treatment (200 μg/mL) samples were analyzed in 1% agarose gel in TAE buffer and then stained with 0.5 μg/mL ethidium bromide for 30 min. SC marks super coiled DNA, while OC marks open circle/nicked circular DNA.

Nme2 is involved in DNA structural changes necessary for the activity of the c-*myc *promoter. It binds to the NHE sequence of the *c-myc *promoter cloned into pUC19 which yields mostly open circle (nicked circular) plasmid [40]. We have considered the possibility that the sponge NmeGp1Sd may already have DNA topoisomerase-like activity similar to the human Nme2. To test this activity, NmeGp1Sd protein was incubated with supercoiled pUC19 plasmid containing the 57-bp *c-myc *NHE sequence. We used topoisomerase I, Nme1 and Nme2 proteins as controls. Control topoisomerase I and human Nme2 cleaved negatively supercoiled plasmid DNA unlike NmeGp1Sd and human Nme1 which did not display this activity (Figure 4).

### The human cell recognizes the sponge Nme as its own

**Figure 5:**
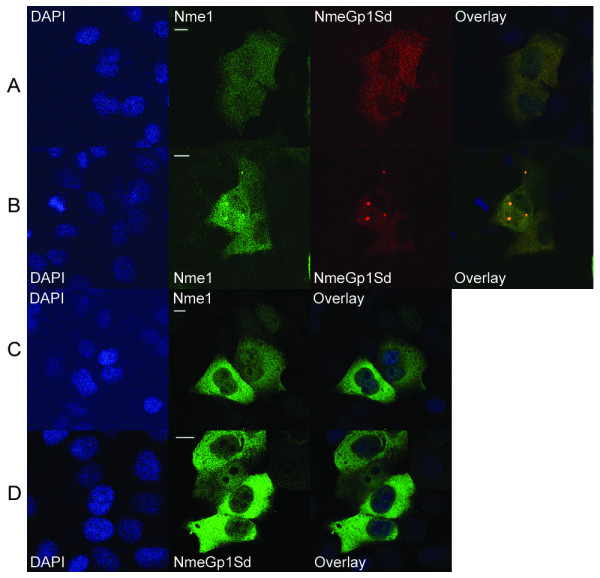
**Subcellular localization of NmeGp1Sd and human Nme1**. A) HEp-2 cells transiently transfected with pEGFPN1-Nme1 (green fluorescence) and pDsRed-NmeGp1Sd (red fluorescence). The signal is visible mainly in the cytoplasm. The overlay (yellow) shows colocalization of the human and sponge homologs. B) HEp-2 cells transiently transfected with pEGFPN1-Nme1 (green fluorescence) and pDsRed-NmeGp1Sd (red fluorescence). The signal is visible mainly in the cytoplasm. The "granum-like" structures are visible in the cytoplasm, and they are formed with both, the human and the sponge homolog. The overlay (yellow) displays the subcellular colocalization of human and sponge homologs. C) HEp-2 cells transiently transfected with pEGFPN1-Nme1. The signal is present in the cytoplasm, but also in the nucleus, although not so prominent. D) HEp-2 cells transiently transfected with pEGFPN1-NmeGp1Sd. The signal is present in the cytoplasm and in the nucleus. Bar = 10 μm.

The objective of testing the localization and function of sponge NmeGp1Sd in well established human *in vitro *systems was to reveal whether the sponge NmeGp1Sd can be recognized as the domestic protein in the human cell and whether it can replace its human homolog. To compare the subcellular localization of human Nme1 and NmeGp1Sd in HEp-2 cells we transiently transfected the cells with pEGFPN1-Nme1 and pDsRedN1-NmeGp1Sd. We analyzed the cells 48 hours post transfection using confocal laser scanning microscopy (Figure 5). The localization of Nme1-GFP was mainly the same as in a previous study [41] in which Nme1 was fused to the C-terminus of GFP (reverse orientation). The signal is mainly present in the cytoplasm, has a punctiform structure with occasional bigger highly fluorescent "granum-like" structures present in the cytoplasm, often, but not exclusively, located adjacent to the nucleus. Nme1 can also very clearly be seen in a portion of the nuclei. The NmeGp1Sd-DsRed reveals the same localization pattern which could be seen in separate figures and merged with human Nme1 (yellow staining). To confirm the NmeGp1Sd nuclear localization we performed additional experiments and transfected HEp-2 cells with pEGFPN1-NmeGp1Sd since the GFP has a brighter fluorescence than the DsRed and is, therefore, easier to analyze. These experiments confirmed the occasional presence of NmeGp1Sd in the nucleus. The fluorescent NmeGp1Sd also seems to form "granum-like" structures and they colocalize with the ones formed by human Nme1. These experiments show that the human and the sponge Nme colocalize - adopt the same distribution in all transfected HEp-2 cells analyzed, at any time-point. These experiments encouraged us to further analyse the interaction of human Nme1 and NmeGp1Sd in human cells.

**Figure 6:**
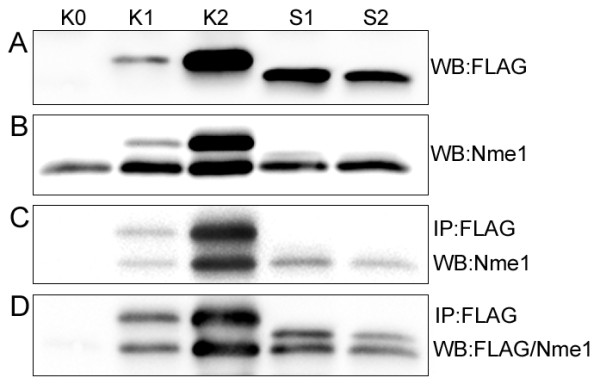
**Nme1/NmeGp1Sd complex formation analysis:** A) Input control: Cell lysates from control K0 (CAL 27 cells stably transfected with pcDNA3 vector), K1 and K2 (CAL 27 cells stably transfected with pcDNA3FLAG/Nme1 construct) and S1 and S2 (CAL 27 cells stably transfected with pcDNA3FLAG/NmeGp1Sd constructs) tested with anti-FLAG antibody. B) Cell lysates from control K0 (CAL 27 cells stably transfected with pcDNA3 vector), K1 and K2 (CAL 27 cells stably transfected with pcDNA3FLAG/Nme1 constructs) and S1 and S2 (CAL 27 cells stably transfected with pcDNA3FLAG/NmeGp1Sd constructs) tested with anti-Nme1 antibody. C) Immunoprecipitation: FLAG/Nme1 (K1 and K2) and FLAG/NmeGp1Sd (S1 and S2) were immunoprecipitated with anti-FLAG M2 affinity gel and immunoblotted with anti-Nme1 antibody. K0-control (clone with "empty" construct). FLAG/Nme1 produces heteromers with exogenous (upper band) and endogenous (lower band) Nme1. FLAG/NmeGp1Sd produces complexes with the endogenous (human) Nme1 while the upper band (exogenous, FLAG/NmeGp1Sd) cannot be seen, since the antibody is specific for human Nme1. D) Immunoprecipitation: FLAG/Nme1 (K1 and K2) and FLAG/NmeGp1Sd (S1 and S2) were immunoprecipitated with anti-FLAG M2 affinity gel and immunoblotted with anti-FLAG and anti-Nme1 antibody. K0-control (clone with "empty" construct). FLAG/Nme1 produces heteromers with exogenous (upper band) and endogenous (lower band) Nme1. FLAG/NmeGp1Sd produces complexes with the endogenous (human) Nme1. The upper band (exogenous, FLAG/NmeGp1Sd) is visible, since it is stained with anti-FLAG antibody.

**Figure 7:**
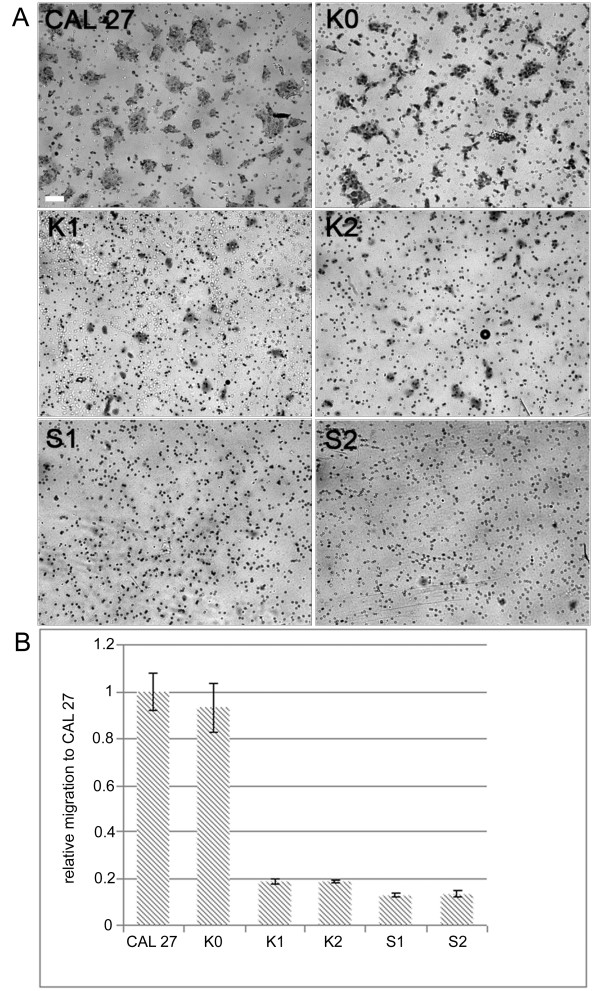
**Migration assay**. A) CAL 27 cells, stable clones transfected with pcDNA3 vector (K0), pcDNA3FLAG/Nme1 (K1 and K2) and pcDNA3FLAG/NmeGp1Sd (S1 and S2) were tested for migration potential in Boyden chambers. The cells were stained with crystal violet. The images were recorded by inverted light microscope. The K and S clones clearly show lower migratory potential compared to untransfected CAL 27 and K0 control cells. Bar = 50 μm. B) The results are presented as a relative number of migrated cells (± SD) compared to CAL 27. The results were produced by ImageJ program measuring the membrane area covered with migrated cells. The results show that S1 and S2 clones as well as K1 and K2 exhibit diminished migration potential compared to CAL 27 and CAL 27 transfected with "empty vector" (K0).

In Western blot analysis a few CAL 27 clones expressing NmeGp1Sd were identified, and two of them (S1 and S2) were chosen for further analysis (Figure 6A and 6B). To test the possible interactions of sponge and human Nme proteins we used the previously produced Nme1 overexpressing CAL 27 clones K1 and K2 [39]. To test if sponge FLAG/NmeGp1Sd forms complexes with endogenous human Nme1, FLAG was immunoprecipitated from cell lysates of FLAG/Nme1 overexpressing clones (K1 and K2), and FLAG/NmeGp1Sd expressing clones (S1 and S2) with anti-FLAG M2 affinity gel. The results of the Western blot analysis with anti-Nme1 or anti-Nme1 and anti-FLAG antibody reveal that FLAG/NmeGp1Sd forms complexes with the endogenous Nme1 (lower band in Figure 6C and 6D). Therefore, we have proved that the human Nme1 recognizes the sponge variant as a partner. The final confirmation of our theory that the human cell recognizes the sponge protein as its own comes from tests in Boyden chambers. The intention of this experiment was to test the migration potential of CAL 27 cells stably expressing sponge NmeGp1Sd, and compare it with the human Nme1 overexpressing cells that show diminished migratory potential compared to control, CAL 27 cells. Figure 7 shows that NmeGp1Sd expressing clones, (as well as human Nme1 overexpressing clones as previously published [39]) have substantially diminished migratory potential. Therefore, NmeGp1Sd suppresses motility of CAL 27 cells. The same result was observed in three independent experiments.

## Discussion

The objective of this study was primarily to determine the structure and biochemical characteristics of the NmeGp1Sd in order to gain a deeper insight into the evolution of metazoan Nme proteins and their functions.

Sponge Group I *Nme *genes are intron-rich and these introns are relatively short. The same has been found for introns in several other sponge genes [42,43] and recently in *A. queenslandica *genome where median intron size is 80 bp [32]. The fourth intron (Figure 1) is likely the most ancient because it is also found in a choanoflagellate Group I *Nme *homolog. We conclude that the ancestral metazoan Group I *Nme *gene was intron-rich and probably had all four introns that are still present in most extant basal metazoan homologs. The ancestral gene structure is also well preserved in vertebrate homologs with three out of four introns present. *D. melanogaster *has only one of the ancestral introns and *C. elegans *lost all ancestral introns and gained two new ones which likely reflect accelerated evolution in these lineages. Analysis of sponge *NmeGp1 *promoters showed that some of the motifs essential for human promoter activity are also present in sponges. We did not find these motifs in the corresponding choanoflagellate promoter under the same search parameters, which indicates a possible change in Nme1 regulation in the metazoan lineage.

The phylogenetic tree (Additional file 2) is generally not well resolved or supported, as in comparable earlier studies [1,2,24]. Nonetheless, it does provide some new evidence on Group I Nme evolution. Group I *Nme *gene duplicates in *N. vectensis *had been proposed to indicate that the split into *Nme1 *and *Nme2 *might have occurred very early in metazoan evolution [1]. Another recent study [24] showed that duplications of Group I *Nme *genes occurred independently more than once in invertebrates, besides well described duplications and diversifications of Group I *Nme *to *Nme1*, *2*, *3 *and *4 *in vertebrate lineage, and that duplication in *N. vectensis *is also an independent event unrelated to the origin of *Nme1 *and *Nme2*. Our results confirm these findings and additionally demonstrate independent duplications in basal metazoans, even within the same lineages (cnidarians and calcareous sponges).

The metastasis suppressor genes *Nme *are highly conserved in opisthokonts. This implies that they have important roles in basic cellular functions. A number of studies revealed several potential biological roles of Nme genes/proteins, but most are not confirmed *in vivo*. The expression of the *D. discoideum *Nme homologues gip17 and guk7.2 is modulated during the developmental phases of cell growth or aggregation due to starvation [44]. In *Drosophila*, Nme/Awd is required for the proper differentiation of many tissues including the brain, eye, and female reproductive system [45]. Nme protein accumulation is coincident with the functional differentiation of multiple epithelial tissues in the developing mouse [18]. These and many other studies demonstrate that Nme proteins have a critical role in differentiation and development - processes that involve cell migration, a prerequisite for metastasis formation [1].

The sponge protein NmeGp1Sd was compared primarily with human Nme1 (and Nme2) for several reasons: i) The NmeGp1Sd is highly similar to vertebrate Nme1/Nme2 in primary structure, ii) human Nme1/Nme2 proteins are the most studied and well characterized Group I Nme members iii) Nme1 is linked to metastasis suppression, but the suppression mechanism is not well understood. We expected that NmeGp1Sd analysis and the discovery of its biochemical features would shed some more light into the mechanisms connecting various Nme1 functions and its antimetastatic activity. A recent study by Domazet-Lošo and Tautz [46] shows that, contrary to what might be intuitively expected, only a subset of cancer genes appeared simultaneously with multicellularity in animals and that the new genetic processes and gene functions that emerged at the basis of the metazoan lineage are major innovations which enabled complex interactions between metazoan cells. Demosponges have only one Group I Nme protein (whereas amniotes have four with potentially different functions), which is probably the ancestral condition for metazoans.

Our results show that the sponge NmeGp1Sd possesses some biochemical characteristics typical for human Nme1 and some typical for human Nme2 protein. Recombinant NmeGp1Sd appears to be predominantly in the hexameric form like human Nme1 protein which was confirmed by gel filtration (Figure 2A and 2B). The sponge NmeGp1Sd and human Nme1 enzymes also have similar levels of kinase activity. Human Nme1 did not display an ability to bind sscDNA, however, human Nme2 can bind sscDNA and showed the same DNA band retardation effect as the sponge homolog. Accordingly we decided to check the possibility that the sponge NmeGp1Sd is functionally more similar to Nme2 than to Nme1. Unlike Nme1, Nme2 is able to cleave *c-myc *NHE sequence [40]. Therefore, we tested whether the sponge NmeGp1Sd also has DNA topoisomerase-like activity and found out that it is not able to cleave *c-myc *NHE sequence. We hypothesize that this was the ancestral condition of the Nme Group I protein before duplications and functional diversifications within the Group I Nme. Sponge NmeGp1 may have retained the multiple functions, while these functions have been partitioned between different vertebrate Group I Nme proteins. Recent studies showed that sponge genomes are comparable to genomes of "higher" complex animals, including vertebrates, in terms of gene number and functional repertoire [32,33]. It was suggested that complex animals differ from their early simple relatives mainly in the more complex regulation of similar sets of genes. From that point of view, it could be speculated that the ability of Nme2 to cleave *c-myc *is a relatively recent addition to the regulatory networks.

Based on this evidence we then asked the following: What is the biological function of NmeGp1Sd in its native environment - the sponge cell? As it can suppress the migration of CAL 27 cells e.g. replaces the human Nme1 it could thus be involved in migration and/or, possibly, adhesion of sponge cells. It has previously been found that sponges possess genes/proteins for many advanced physiological processes usually linked to more complex metazoan phyla [31,47]. Migration of cells in complex "higher" animals is limited to specific processes such as embryonic development and immune response and is closely controlled. Cells are anchored in the developed tissues they belong to and do not migrate if not stimulated (with the exception of cells undergoing malignant transformation). The anchorage and migration of cells depends on a vast number of molecules such as cadherines, integrins, fibronectin and collagen. Although sponges do not possess true tissues and organs, they do possess simple varieties of the mentioned proteins [48,49]. Furthermore, they have complex pelagobenthic life cycles which include development to a larval phase and metamorphosis to sedentary adult form [50]. Cell migration is present in sponges in three different processes: regeneration, larval development and the movement of amoeboid cells through the adult mesohyl; the space between the external and internal cell layers which is composed of galectin, collagen, fibronectin-like molecules and dermatoponin [51]. Although the mesohyl is not a homogenous, organized structure, it resembles a primitive extracellular matrix (ECM). Therefore, both the movement of amoeboid cells through the mesohyl in sponges and the movement of cells through ECM in vertebrates may originate from the same ancient precursor process present in the metazoan last common ancestor. From an evolutionary point of view, tumors likely developed together with the evolution of tissues and organs. It is not yet defined which biochemical functions of Nme are crucial for its metastasis suppressor activity. It is, however, to be expected that its main biological function (besides the well defined NDPK function), would not be to suppress metastasis in "higher" Metazoa but to control normal physiological processes (adhesion, migration) which, if left without control, could lead to metastasis formation. As sponges do not possess tissues and organs they are probably also incapable of forming tumors. The demonstration of the fact that the sponge NmeGp1Sd can replace human Nme1 in human tumor cells encourages us to propose that the NmeGp1 (at least Nme1) function responsible for regulating adhesion/migration of cells was established in the metazoan ancestor well before the Cambrian explosion i.e. before the appearance of diverse groups of multicellular Metazoa. Since tumors likely started developing in parallel with the development of true tissues and organs, this ancient function of Nme1 was then adapted to act in a new highly significant role - the suppression of metastasis.

## Conclusions

In conclusion, our study emphasizes three major points: i) the single sponge Group I *Nme *gene and Nme protein of *Suberites domuncula *probably reflects the characteristics of the ancestral Group I Nme gene/protein, that existed prior to the duplication of this group in vertebrates and other lineages; ii) NmeGp1Sd possesses many biochemical properties of the most recent Group I variant, the Nme1 human homolog; iii) NmeGp1Sd interacts with human Nme1 and can replace it in some biological functions which are usually associated with "higher" metazoans, specifically in metastasis suppression. Therefore, we presume that the Group I Nme gene/protein of the metazoan last common ancestor was structurally and functionally similar to the multifunctional enzyme it is today.

## Methods

### Sequence and phylogenetic analyses

For genomic DNA preparation, the specimens of *S. domuncula *were cut into pieces, frozen in liquid nitrogen, ground to fine powder from which total DNA was isolated using the Genomic DNA Purification kit (QIAGEN). Sponge *NmeGp1Sd *gene was amplified by PCR, using KOD XL DNA polymerase (Novagen) and primers specific for the 5'-(GCTTTTCTGTGTGTGGAGCTT) and 3'-(TCCCTAAACCAAAAAGTTACTCAT) ends of the cDNA coding sequence [GenBank:AY764256]. The amplified fragment was separated on a 0.8% agarose gel, purified and sequenced. The reverse specific primer 5'- CATGGCAACCATCTTGAAGCC -3' was used in conjunction with the vector specific primer T3 to amplify the 5' upstream region from translation start of the *nmeGp1Sd *gene on the genomic library from *S. domuncula *[52].

Nucleotide sequences were stored and analyzed using Lasergene (DNAStar, Madison, WI). Multiple sequence alignments (MSA) were performed with CLUSTALX [53]. The exact position and the phase of each intron were verified by manual inspection. MEGA4 [54] was used to construct and bootstrap phylogenetic trees.

The homolog of the *NmeGp1Sd *gene from the sponge *Amphimedon queenslandica *was identified in the genome (contig13509) available at http://spongezome.metazome.net/cgi-bin/gbrowse/amphimedon/[32]. Homology searches and sequence retrievals were done using BLAST (NCBI, NIH, Bethesda, MD, USA: http://www.ncbi.nlm.nih.gov). NmeGp1 sequences from sponges *Leucetta *and *Sycon *were identified by blastn with *nmeGp1Sd *sequence against NCBI's dbEST limited to Porifera. Hamming clustering method for TATA signal prediction in eukaryotic genes was used for identifying TATA-box http://www.itb.cnr.it/sun/webgene/[55]. TFSEARCH http://www.cbrc.jp/research/db/TFSEARCH.html and TRANSFAC database [56], with a default threshold score of 85 were used for searching -400 bp 5' flanking region for possible transcription factors binding sites.

### Plasmid constructions

We used the same set of primers as for *NmeGp1Sd *gene to perform PCR reactions on the *S. domuncula *cDNA library. The PCR generated cDNA fragment was cloned into pBluescript vector (Stratagene). The cDNA for NmeGp1Sd was recloned into pET15b (Novagen) using PCR into *Bam*HI (5'-TTCCCGGATCCAAAAAGTTACTCATAGATC-3') and *Nde*I (5'-GCTTTATACATATGACAACCGAGCGTACC-3') restrictions sites of pET15b expression vector downstream from the thrombin cleavage site. For colocalization assay, the full-length coding sequence *NmeGp1Sd *was cloned in frame into pEGFPN1 and pDsRedN1 expression vectors (Clontech, USA). The fragment of 541 bp was amplified using 5'-GCTTTCTCGAGATGACAACCGAGCGTACC-3' and 5'-GTAGACGGATCCTTCTCATAGATCCATTGCTG-3' primers. The PCR product was digested with *Xho*I and *Bam*HI, and ligated with the *Xho*I/*Bam*HI sites of pEGFP-N1 and pDsRedN1 expression vectors. The pEGFPN1-Nme1 was a kind donation of Dr. Marie-Lise Lacombe, Faculty of Medicine St. Antoine, Paris, France. NmeGp1Sd cDNA was amplified using a forward primer containing the FLAG sequence (F: 5'-GTCTAGGGATCCACGAGATGGACTACAAGGACGACGACGATAAGATGACAACCGAGCGT-3') and the reverse primer (R: 5'-CTAGACGAATTCTTATTACTCATAGATCCATTGCTGTTCTGTGGG-3'). The PCR product was cloned into the *Bam*HI/*Eco*RI sites of eukaryotic expression vector pcDNA3 (Invitrogen).

### Protein expression and purification

NmeGp1Sd was overproduced in *Escherichia coli *strain BL21 tagged with six histidine residues at the N-terminus and purified to homogeneity from bacterial lysates using nickel affinity chromatography. *E. coli *strain BL21 harboring the plasmid construct was grown to 0.6 at OD_600 _and induced with 0.8 mM IPTG for 3 hours at 37°C. Cells were incubated 30 min on ice in lysis buffer (50 mM Tris HCl, 300 mM NaCl, 10 mM imidazole and 1mg/mL lysozyme) and sonicated 8 × 30 sec (50% of the full power). After centrifugation (12000 rpm) for 25 min at 4°C, the supernatant was applied onto nickel-charged agarose column (Qiagen). Histidine tagged proteins bound to nickel-affinity resins were eluted with 500 mM imidazole. NmeGp1Sd protein was applied to a PD-10 desalting gel filtration column (Sephadex, GE Healthcare) equilibrated with 25 mL of Nm23 buffer [57] (20 mM HEPES at pH 7.9, 5 mM MgCl_2_, 0.1 mM EDTA, 2 mM DTT and 0.1 M KCl) and then eluted with 3.5 mL of Nm23 buffer. The polyhistidine tag was released from the fusion protein by cleaving with thrombin (Sigma) (2 U/40 μg of fusion protein) for 16 hours at room temperature. The human Nme1 protein was a kind donation of Dr. Ioan Lascu (Institut de Biochemie et Genetique Cellulaires-IBGC, Bordeaux, France).

### Protein cross-linking with glutaraldehyde

Oligomerization of the recombinant protein NmeGp1Sd was performed in PBS buffer as described by Kim et al. [38] for human Nme1 proteins with modified conditions: eight μg of sponge NmeGp1Sd was pre-incubated in PBS at room temperature and 25 mM of glutaraldehyde was added to initiate the cross-linking. Following 5 min incubation, the reaction was quenched with 0.2 M Tris-HCl, pH 7.5, for 15 min at room temperature. The reaction product was subjected to 12.5% SDS-PAGE and visualized by protein staining with Coomassie brilliant blue.

### Gel filtration chromatography

Recombinant human Nme1 and sponge NmeGp1Sd were loaded onto Bio-Sil SEC 250 gel-filtration columns (300 mm × 7.8 mm) and eluted with Nm23 buffer [57] at a flow rate of 0.5 mL/min (BioLogic Duo Flow, BIO-RAD). Peak fractions were used for NDP kinase assay.

### DNA-binding assay

The DNA-binding activity of the NmeGp1Sd protein was assayed *in vitro *as described [58]. Reactions contained 30 nM of single-stranded circular DNA from bacteriophage ϕX174 (NEB). The amount of purified NmeGp1Sd protein is indicated (Figure 3). Human proteins Nme1 and 2 were used as controls. The reaction mixture was incubated in 20 μl of buffer containing 40 mM Tris-acetate (pH 7.5) and 12 mM MgCl_2_. After 15 min of incubation at 37°C, 4 μL of 40 mM Tris-acetate (pH 7.5) containing 50% glycerol and 0.01% bromophenol blue was added and the products were immediately subjected to gel electrophoresis at 3 V/cm in 0.6% agarose for 3 hours using 40 mM Tris-acetate (pH 7.5) and 1 mM EDTA as running buffer.

### NDP kinase assay

Enzyme-coupled Reaction-NDPK activity was measured using a coupled pyruvate kinase-lactate dehydrogenase assay [57,59]. Five hundred μL of reaction mixtures were incubated in quartz cuvettes at room temperature in the presence of ATP as phosphate donor and dTDP as phosphate acceptor. The final concentrations were as follows: 50 mM Tris-HCl pH 7.4, 75 mM KCl, 5 mM MgCl_2_, 1 mM phosphoenolpyruvate, 0.1 mg/mL NADH, 1 mM ATP, 0.2 mM dTDP, 2 U/mL of pyruvate kinase, 2.5 U/mL of lactate dehydrogenase, and 1 mg/mL bovine serum albumin. All reagents and enzymes were purchased from Sigma. Reactions were initiated by the addition of 50 ng of NmeGp1Sd enzyme and activity was monitored in an Ultrospec^® ^2100 *pro *(Amersham Pharmacia Biotech, USA) measuring the decrease in absorbance at 340 nm. The control reactions omitting NmeGp1Sd produced minor rates but were subtracted from the NmeGp1Sd values. The same reaction was performed with human Nme1 enzyme.

### Plasmid cleavage assays

We cloned the double stranded 57-bp NHE (nuclease hypersensitive element) of the *c-myc *promoter (5'-GATCCCCAGTCTCCTCCCCACCTTCCCCACCCTCCCCACCCTCCCCATAAGCGAATT-3') insert into pUC19 plasmid. Recombinant plasmid pUC19-NHE (500 ng) was incubated with 500 ng of NmeGp1Sd protein. The reaction was assembled in 50 mM KCl, 10 mM MgCl_2_, 50 mM Tris HCl pH 7.5, 0.5 mM DTT, 30 μg/mL BSA and incubated 30 min at 20°C. The reaction was terminated with 1% SDS, 10 mM EDTA and proteinase K treatment (200 μg/mL) for 30 min at 55°C. Topoisomerase I (Gibco BRL), Nme1 and Nme2 proteins were used as controls and were processed in the same conditions. Samples were analyzed in 1% agarose gel in TAE buffer and than stained with 0.5 μg/mL ethidium bromide for 30 min.

### Cell culture

Human head and neck tumor cell line HEp-2 (squamous cell carcinoma of the larynx) was obtained from ATCC, while CAL 27 (squamous cell carcinoma of the tongue, ATCC) were a kind donation of Dr. Jeannine Gioanni, Centre Antoine Lacasagne, Nice, France. Both cell lines were cultured in Dulbecco's modified Eagle medium (DMEM, Invitrogen) supplemented with 10% fetal bovine serum (FBS, Invitrogen), 2 mM glutamine, 100 U/mL penicillin and 100 μg/mL streptomycin in humidified chamber with 5% CO_2 _at 37°C.

### Transient transfections and laser scanning confocal microscopy

Twenty four hours before transfection 3 × 10^4 ^HEp-2 cells were seeded on eight-well culture slides (BD-Falcon) to obtain 60% confluence. Each chamber was transfected with 1 μg of plasmid DNA: pEGFPN1-Nme1, pEGFPN1-NmeGp1Sd, or cotransfected with pDsRedN1-NmeGp1Sd and pEGFPN1-Nme1 (0.5 μg of each construct) using Lipofectamine reagent (Invitrogen) according to the manufacturer's instructions. Forty eight hours post transfection the cells were washed with PBS (phosphate-buffered saline, pH 7.5), fixed in 4% formaldehyde, and mounted in mounting medium (DAKO) supplemented with 1 μg/mL 4,6-diamino-2-phenylindole (DAPI) (Sigma) for nuclear staining.

Fluorescent images were obtained by Leica TCS SP2 AOBS laser scanning confocal microscopy equipped with HCX PL APO λ-Blue 63 × 1.4 objective. GFP was excited by 488 nm laser line, DsRed using 543 nm, and DAPI using 405 nm laser line.

### Preparation of stably transfected CAL 27 clones

Stably transfected cell lines were prepared as described previously [60]. In brief, CAL 27 cells were transfected using Lipofectamine reagent (Invitrogen) with pcDNA3FLAG/NmeGp1Sd and pcDNA3 as control. Post transfection the cells were resuspended and incubated in DMEM supplemented with geneticin (Sigma) until development of resistant colonies. Positive clones were screened by Western blotting, propagated and frozen until further usage. The clones with overexpressing FLAG/Nme1 (K1 and K2) have been formed in the exact same way and are the same ones used in [39].

### Immunoprecipitation and Western blotting

For detection of Nme1/NmeGp1Sd complex formation 1 × 10^6 ^CAL 27 cells stably transfected with "empty" vector (K0), as well as CAL 27 cells stably expressing FLAG/NmeGp1Sd (S1 and S2) and FLAG/Nme1 expressing clones (K1 and K2) were seeded on 6-well plates overnight. Immunoprecipitation of FLAG/Nme1 was performed using anti-FLAG M2 affinity gel (Sigma) according to manufacturer's instructions.

The proteins were separated by SDS-PAGE and electrotransfered to an Imobilon-PSQ membrane (Milipore). The membranes were incubated with anti-Nme1 antibody (Beckton-Dickinson) or with anti-FLAG M2 antibody (Sigma) and Nme1 antibody for detection of complex formation. Protein bands were visualized using Chemiluminiscence blotting substrate-POD (Roche) on Lumi-Film-Chemiluminiscent Detection Film (Roche).

### Migration in Boyden chambers

Boyden chamber assay was done as described before [39]. Briefly, two six well plates were seeded with 3 × 10^5 ^cells (CAL 27, CAL 27 stably transfected with "empty" vector, CAL 27 constitutively overexpressing FLAG/Nme1 (K1 and K2) and CAL 27 constitutively expressing NmeGp1Sd (S1 and S2)) overnight. After attachment the cells were starved in serum free medium for 24 hours at 37°C in a humidified chamber. After starvation the cell suspension (4 × 10^5 ^cells) was added to the upper chamber of Cell Culture Inserts (Beckton-Dickinson) and allowed to settle down for 10 min. DMEM supplemented with 10% FBS served as a chemoattractant and was added to the lower chamber. Cells were allowed to migrate for 20 hours. Nonmigratory cells were detached with cotton swabs, membranes were fixed in 4% formaldehyde, stained with 0.1% crystal violet, cut out from the inserts, mounted in mounting medium (DAKO) and analyzed by light microscopy. The migrated cells were analyzed at 400 × magnitude. Three images per membrane were acquired and analyzed in ImageJ program http://rsbweb.nih.gov/ij/index.html which measured the area of the membrane covered with migrated cells.

## Authors' contributions

DP carried out the molecular genetic studies, biochemical assays and the sequence analysis. MHB and RB carried out colocalization, immunoprecipitation and migration experiments. MD carried out stable clone preparation. AM participated in the biochemical assays and MH performed phylogenetic analysis. HĆ conceived and oversaw the project, participated in the sequence analysis and molecular genetic studies. HĆ, MHB, DP and MH wrote the manuscript. All authors read and approved the final manuscript.

## Supplementary Material

Additional file 1 **Promoter regions**. The structure of *NmeGp1 *promoter regions from sponges *S. domuncula *and *A. queenslandica*. The most plausible putative binding sites for transcription factors identified by TFSEARCH are marked. Motifs shared with human *Nme1 *promoter region are boxed. Arrows denote the orientation of motifs. TSS - transcription start site. Click here for file

Additional file 2 **Maximum parsimony phylogenetic tree of Group I Nme members**. Bootstrap values inferred from 1000 replicates are shown next to the branches (maximum parsimony/neighbour joining support). Accession numbers of sequences used are given in brackets after species names. Click here for file

Additional file 3 **Multiple sequence alignment of NmeGp1 proteins**. Human (Nme1 and Nme2), choanoflagellate *Monosiga brevicollis *(NmeGp1Mb) and sponges *Suberites domuncula *(NmeGp1Sd), *Amphimedon queenslandica *(NmeGp1Aq), *Sycon raphanus *(NmeGp1SrA and NmeGp1SrB) and *Leucetta chagosensis *(NmeGp1LcA and NmeGp1LcB) proteins were aligned. Enzyme active site amino acids and amino acids necessary for the association of subunits on the hexamer are conserved and marked with *. Click here for file
