# Supplementary material for: Sponge non-metastatic Group I Nme gene/protein - structure and function is conserved from sponges to humans
Source: BMC Evol Biol. 2011 Apr 1;11:87. doi: 10.1186/1471-2148-11-87 (PMC3078890; doi:10.1186/1471-2148-11-87)
Supplement: Additional file 1 — Promoter regions. The structure of NmeGp1 promoter regions from sponges S. domuncula and A. queenslandica. The most plausible putative binding sites for transcription factors identified by TFSEARCH are marked. Motifs shared with human Nme1 promoter region are boxed. Arrows denote the orientation of motifs. TSS - transcription start site. [file 1471-2148-11-87-S1.PDF]

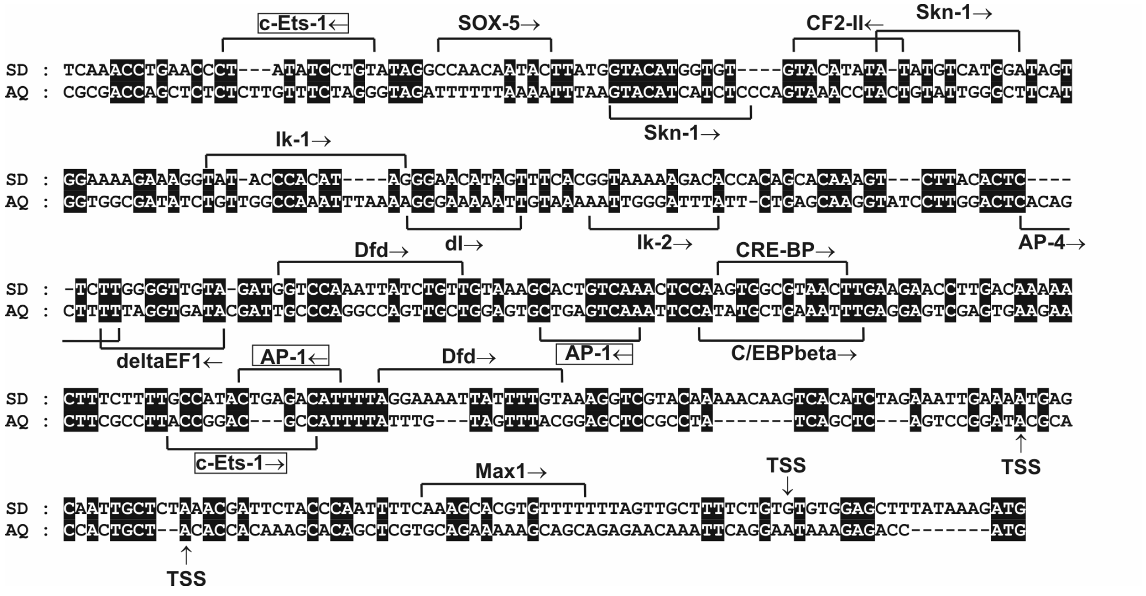

Promoter regions. The structure of *NmeGp1* promoter regions from sponges *S. domuncula* and *A. queenslandica.* The most plausible putative binding sites for transcription factors identified by TFSEARCH are marked. Motifs shared with human *Nme1* promoter region are boxed. Arrows denote the orientation of motifs. TSS - transcription start site.
