# Supplementary material for: Sponge non-metastatic Group I Nme gene/protein - structure and function is conserved from sponges to humans
Source: BMC Evol Biol. 2011 Apr 1;11:87. doi: 10.1186/1471-2148-11-87 (PMC3078890; doi:10.1186/1471-2148-11-87)
Supplement: Additional file 2 — Maximum parsimony phylogenetic tree of Group I Nme members. Bootstrap values inferred from 1000 replicates are shown next to the branches (maximum parsimony/neighbour joining support). Accession numbers of sequences used are given in brackets after species names. [file 1471-2148-11-87-S2.PDF]

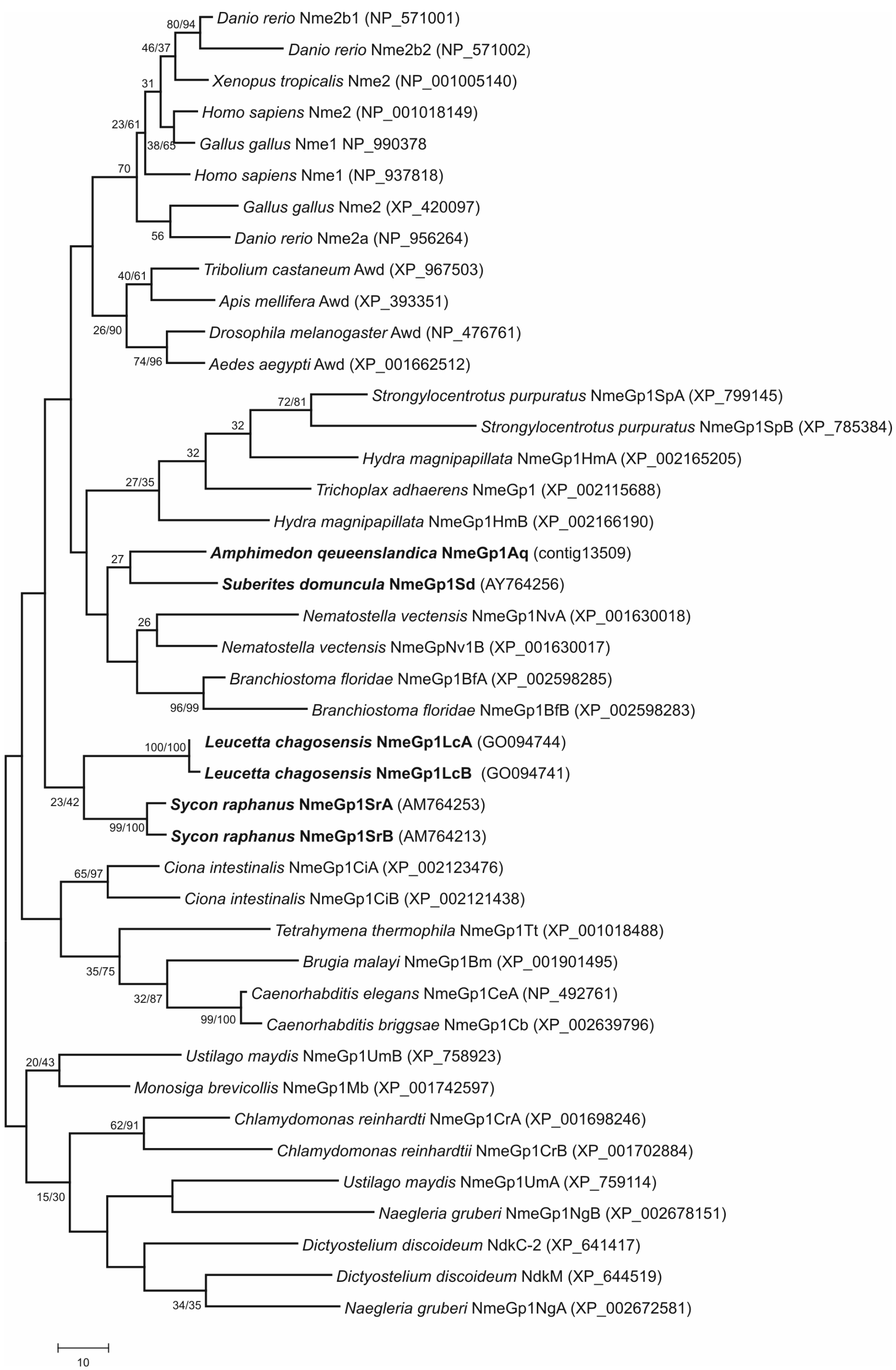

Maximum parsimony phylogenetic tree of Group I Nme members. Bootstrap values inferred from 1000 replicates are shown next to the branches (maximum parsimony/neighbour joining support). Accession numbers of sequences used are given in brackets after species names.
