# Supplementary material for: Sponge non-metastatic Group I Nme gene/protein - structure and function is conserved from sponges to humans
Source: BMC Evol Biol. 2011 Apr 1;11:87. doi: 10.1186/1471-2148-11-87 (PMC3078890; doi:10.1186/1471-2148-11-87)
Supplement: Additional file 3 — Multiple sequence alignment of NmeGp1 proteins. Human (Nme1 and Nme2), choanoflagellate Monosiga brevicollis (NmeGp1Mb) and sponges Suberites domuncula (NmeGp1Sd), Amphimedon queenslandica (NmeGp1Aq), Sycon raphanus (NmeGp1SrA and NmeGp1SrB) and Leucetta chagosensis (NmeGp1LcA and NmeGp1LcB) proteins were aligned. Enzyme active site amino acids and amino acids necessary for the association of subunits on the hexamer are conserved and marked with *. [file 1471-2148-11-87-S3.PDF]

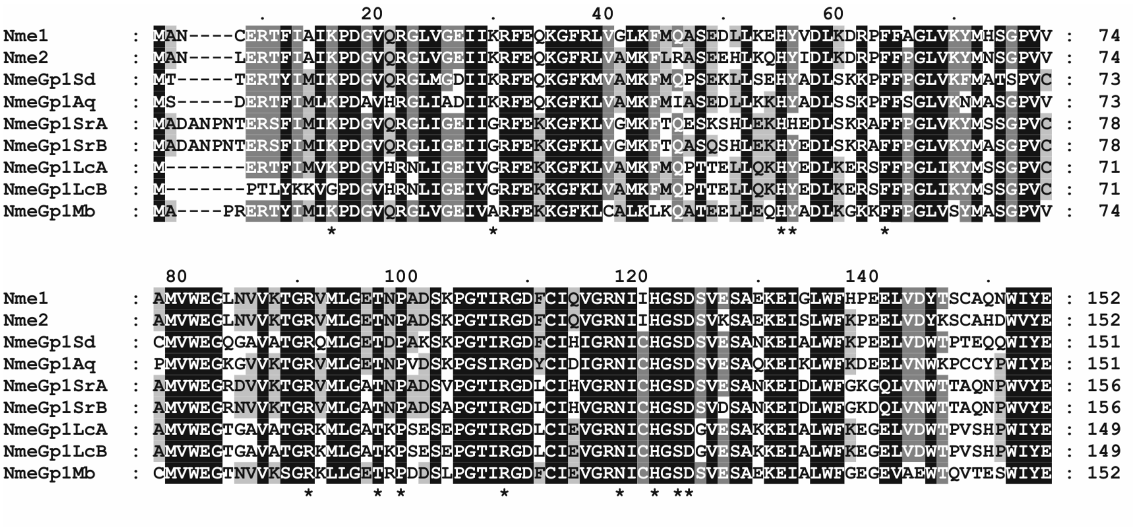

Multiple sequence alignment of NmeGp1 proteins. Human (Nme1 and Nme2), choanoflagellate *Monosiga brevicollis* (NmeGp1Mb) and sponge *Suberites domuncula* (NmeGp1Sd), *Amphimedon queenslandica* (NmeGp1Aq), *Sycon raphanus* (NmeGp1SrA and NmeGp1SrB) and *Leucetta chagosensis* (NmeGp1LcA and NmeGp1LcB) proteins are aligned. Enzyme active site amino acids and amino acids necessary for the association of subunits in the hexamer are marked with *.
